# Memory-Efficient Analysis of Dense Functional Connectomes

**DOI:** 10.3389/fninf.2016.00050

**Published:** 2016-11-29

**Authors:** Kristian Loewe, Sarah E. Donohue, Mircea A. Schoenfeld, Rudolf Kruse, Christian Borgelt

**Affiliations:** ^1^Department of Neurology, Otto-von-Guericke UniversityMagdeburg, Germany; ^2^Department of Computer Science, Otto-von-Guericke UniversityMagdeburg, Germany; ^3^Leibniz Institute for NeurobiologyMagdeburg, Germany; ^4^Center for Cognitive Neuroscience, Duke UniversityDurham, NC, USA; ^5^Kliniken SchmiederAllensbach, Germany

**Keywords:** functional connectivity, dense connectome analysis, resting-state fMRI, big data, graph theoretical analysis

## Abstract

The functioning of the human brain relies on the interplay and integration of numerous individual units within a complex network. To identify network configurations characteristic of specific cognitive tasks or mental illnesses, functional connectomes can be constructed based on the assessment of synchronous fMRI activity at separate brain sites, and then analyzed using graph-theoretical concepts. In most previous studies, relatively coarse parcellations of the brain were used to define regions as graphical nodes. Such parcellated connectomes are highly dependent on parcellation quality because regional and functional boundaries need to be relatively consistent for the results to be interpretable. In contrast, dense connectomes are not subject to this limitation, since the parcellation inherent to the data is used to define graphical nodes, also allowing for a more detailed spatial mapping of connectivity patterns. However, dense connectomes are associated with considerable computational demands in terms of both time and memory requirements. The memory required to explicitly store dense connectomes in main memory can render their analysis infeasible, especially when considering high-resolution data or analyses across multiple subjects or conditions. Here, we present an object-based matrix representation that achieves a very low memory footprint by computing matrix elements on demand instead of explicitly storing them. In doing so, memory required for a dense connectome is reduced to the amount needed to store the underlying time series data. Based on theoretical considerations and benchmarks, different matrix object implementations and additional programs (based on available Matlab functions and Matlab-based third-party software) are compared with regard to their computational efficiency. The matrix implementation based on on-demand computations has very low memory requirements, thus enabling analyses that would be otherwise infeasible to conduct due to insufficient memory. An open source software package containing the created programs is available for download.

## 1. Introduction

Graph-based analysis of dense connectomes allows for spatially precise mapping of fMRI-based functional connectivity patterns but is associated with considerable computational demands (van den Heuvel et al., 2008; Hayasaka and Laurienti, 2010; de Reus and Van den Heuvel, 2013; Fornito et al., 2013). As a result, some previous studies have been conducted at reduced spatial resolution (Buckner et al., 2009; Valencia et al., 2009; Zuo et al., 2012). However, most previous studies have not used dense connectomes at all, neither at the full nor at a reduced resolution. Instead, relatively coarse parcellations (or multiple regions of interests) were used to define network nodes (e.g., Salvador et al., 2005; Achard et al., 2006; Supekar et al., 2008; Fair et al., 2009; He et al., 2009; Supekar et al., 2009; Dosenbach et al., 2010; Fornito et al., 2011; Schoonheim et al., 2012; Agosta et al., 2013; Brier et al., 2014; Suo et al., 2015; Rocca et al., 2016). While the analysis of such parcellated connectomes offers many advantages and led to impactful findings, their spatial sensitivity is rather limited, as the use of region-level nodes typically involves the aggregation of fMRI time series from the incorporated voxels, at the cost of more detailed spatial information (Wang et al., 2010; Scheinost et al., 2012; Stanley et al., 2013). Such analyes are thus highly dependent on parcellation quality because regional and functional boundaries need to be relatively consistent to obtain meaningful results (Smith et al., 2011, 2013; Zuo and Xing, 2014; Jiang et al., 2015; Jiang and Zuo, 2016). In this regard, the suitability of a given parcellation also depends on the application because functional boundaries may vary between individuals (Biswal et al., 2010; Kelly et al., 2012), in the context of different tasks (Lohmann et al., 2016; Mišić and Sporns, 2016), or in association with dysfunction and disease (Matthews and Hampshire, 2016).

Dense connectomes, in contrast to parcellated connectomes, are less prone to these problems because the parcellation inherent to the data is used to define graphical nodes, so that the mixing of multiple, potentially dissimilar signals is avoided. To make the analysis of dense connectomes more computationally efficient, some efforts have recently been directed toward acceleration, e.g., through parallel computing (based on multi-core CPUs (Tomasi and Volkow, 2011; Loewe et al., 2014), GPUs (Wang et al., 2013), Intel® Xeon Phi™ coprocessors (Wang et al., 2015), specialized vector hardware (Minati et al., 2014), or CPU instruction set extensions (Loewe et al., 2014)), or alternative measures of internodal association (Loewe et al., 2014; Minati et al., 2014).

By focusing on time efficiency, however, memory- and storage-related aspects are sometimes overlooked. With high-resolution data, a single connectivity matrix can easily surpass the available main memory of most computers. For example, at an isometric resolution of 2 mm, about 200,000 gray matter voxels exist in MNI template space. Using 4 bytes per element, the corresponding voxel-level connectivity matrix, or dense connectome (Van Essen and Ugurbil, 2012), would thus require about 149 GiB. Of course, there are some straightforward ways to reduce memory usage. As a side effect of the recently introduced grayordinate space, all gray matter can be represented by about 100,000 grayordinates because cortical data are modeled in surface space (Glasser et al., 2013), so that a grayordinate-based dense connectome would require only about 37.3 GiB. Due to matrix symmetry, it would further be sufficient to store only the upper or lower triangular part of a functional connectivity matrix, reducing memory occupancy by about 50%. Although the memory required to explicitly store connectivity matrices can be reduced in this way, they still remain too large for many applications, especially when considering high-resolution data or analyses across multiple subjects or conditions.

Here, we propose an object-based matrix representation that achieves a very low memory footprint by computing matrix elements *on demand* instead of explicitly storing them. In doing so, the memory required for a dense connectome reduces to the amount needed to store the underlying time series data. Even for data that exhibit a very high temporal resolution, this approach allows for immense reductions in memory requirements.

## 2. Materials and methods

In the literature on functional connectivity, the terms matrix, graph, and connectome are often used somewhat interchangeably so that the exact meaning is often dependent on the context. Typically, based on a set of separate brain sites defined as nodes, pairwise statistical associations between the nodes' corresponding time series are used to derive a functional connectivity matrix. From a graph-theoretical perspective, this matrix can also be regarded as an undirected weighted graph or network, which is often binarized based on a connectivity threshold. Formally, an undirected binary graph *G*_*B*_ consists of two sets, a set of nodes and a set of pairwise internodal connections, or edges. *G*_*B*_ can be represented as an adjacency matrix **B**, where *b*_*ij*_ = 1 indicates that an edge exists between the two nodes *i* and *j*. The term connectome, originally defined in terms of structural brain connectivity (Sporns et al., 2005; Sporns, 2011), is now commonly used in a more ambiguous fashion often referring to brain connectivity graphs or networks of any kind (e.g., binary or weighted graphs, derived from structural, functional, or effective connectivity) and on multiple scales (e.g., microscopic, macroscopic, or at the systems level).

In the context of MRI, one can further distinguish between dense, i.e., voxel- or grayordinate-based, and parcellated connectomes (see e.g., Akil et al., 2011; Marcus et al., 2011; Glasser et al., 2013). In the latter, nodes are not individual voxels or grayordinates, but rather parcels, each of which typically comprises many voxels or grayordinates. Note that in this context dense connectomes could also be viewed as parcellated connectomes with atomic parcels. Note also that the use of the term “dense” here is distinct from its typical use to denote “dense” matrices as opposed to “sparse” matrices, even though a dense connectome can be represented by a dense connectivity matrix.

In this article, we describe our work with dense connectomes in mind. Of course, the proposed methods could be applied to parcellated connectomes as well, but, due to a much smaller number of nodes, such analyses are typically less challenging regarding computational efficiency.

The remainder of this section consists of three parts. We begin with a discussion of possible implementation variants for an object-based matrix representation, focusing on memory requirements and computation time. Next, programs for an example application (degree computation starting out from nodal time series data) based on the proposed matrix object implementation variants are described. The last part deals with the comparison of these programs with each other and with four additional programs based on external tools to assess the computational performance of the different implementation variants with respect to both time and memory efficiency.

### 2.1. Matrix representation

Essentially, to design an object-based representation of a functional connectivity matrix (dense connectome), only three methods are required for interfacing with the object: a constructor (to create an object), an accessor (to access matrix elements), and a destructor (to free the resources acquired by the object during its lifetime when the object is destroyed). Let us consider the expected memory requirements and computation time of three storage schemes for such an object, which we will refer to as *full-stored, half-stored*, and *on-demand*.

#### 2.1.1. Memory requirements

In the full-stored scheme, upon object construction all pairwise internodal connectivity values are precomputed based on the nodes' corresponding time series data and the full connectivity matrix is stored in main memory. This requires memory in the order of *N*^2^ + *NT*, where *N* is the number of nodes and *T* is the number of points in time for storing the full matrix and the time series data.

In the half-stored scheme, taking advantage of matrix symmetry, only the upper (or lower) triangular elements of the matrix are stored in order to save memory. This requires memory in the order of *N*(*N* − 1)/2 + *NT*.

In the on-demand scheme, a matrix element is computed on demand, i.e., once that specific element is accessed. This requires memory in the order of *NT* (the amount of memory needed for the underlying time series data).

Note that in the full- and half-stored schemes the time series data are needed during object construction only. After that, the memory required for the time series data could be deallocated if not otherwise needed. In contrast, in the on-demand scheme, the time series data need to be kept in memory until the object is destroyed. Nevertheless, because in the considered application domain *N* is much greater than *T*, the on-demand scheme provides superior memory efficiency compared to both other schemes.

#### 2.1.2. Computation time

To speed up execution, we consider (1) parallelization through concurrent computations on multiple CPU cores based on multi-threading, (2) data locality optimization (within each thread) through tiling, (3) vectorization through SIMD^1^ instruction set extensions (within each thread), (4) using the tetrachoric correlation coefficient *r*_*t*_ instead of Pearson's *r* to estimate functional connectivity (the latter being used by the majority of previous studies).

##### 2.1.2.1. Multi-threading

To benefit from parallelization by dividing the computations across multiple cores, parallelization through multi-threading (based on Pthreads) is employed in the half-stored scheme so that each of *k* threads computes (approximately) *E*/*k* matrix elements, where *E* = *N*(*N* − 1)/2 is the total number of matrix elements. For the on-demand scheme, parallelization through multi-threading cannot be implemented by the object for obvious reasons. However, it can be implemented by the client or application code, i.e., the code that uses the object (at the cost of additional programming efforts).

##### 2.1.2.2. Data locality optimization

To benefit from data locality, the order in which the matrix elements are computed can be determined in an attempt to minimize the number of cache misses. For the half-stored scheme, we adopted a cache-oblivious tiling (COBL) approach to achieve this (Frigo et al., 1999; Prokop, 1999). Implementation details can be found in Loewe et al. (2014). For the on-demand scheme, data locality optimization has not been attempted, since, on the part of the matrix object, information on the order of accesses is not available in advance. Note that such information is often available on the part of the application code, but inter-dependencies between application code and implementation details of the matrix object should be avoided because the object implementation could change in the future. However, we discuss a special case below (see Section 2.1.3).

##### 2.1.2.3. Vectorization

An fMRI data set can be represented by a data matrix ${\text{X}}^{N\times T}=\left(x_{ik}\right)$, where 1 ≤ *i* ≤ *N* and 1 ≤ *k* ≤ *T*. By *x_i_* = (*x*_*i*1_, *x*_*i*2_, ⋯, *x*_*iT*_), i.e., the *i*th row of X, we denote the time series of the *i*th node. Using Pearson's *r* as a connectivity measure, the sample correlation matrix is given by ${\text{R}}^{N\times N}=\left(r_{ij}\right)$, where each matrix element can be computed as the mean of the products of the standard scores using

$$r_{ij}=\frac{1}{T-1}\overset{T}{\underset{k=1}{\sum }}\left(\frac{x_{ik}-{\bar{x}}_{i}}{s_{x_{i}}}\right)\left(\frac{x_{jk}-{\bar{x}}_{j}}{s_{x_{j}}}\right)=\frac{z_{i}\cdot z_{j}}{T-1},$$

where ${\bar{x}}_{i}$, *s*__*x*__*i*__, and *z_i_* (with *z_i_* = (*z*_*i*1_, *z*_*i*2_, …, *z*_*iT*_) and $z=\frac{x-\bar{x}}{s_{x}}$) are the sample mean, the corrected sample standard deviation, and the vector of standard scores corresponding to *x_i_*, respectively. This equation can be rearranged to

$$r_{ij}=\overset{T}{\underset{k=1}{\sum }}\left(\frac{x_{ik}-{\bar{x}}_{i}}{\sqrt{\left(T-1\right)s_{x_{i}}^{2}}}\right)\left(\frac{x_{jk}-{\bar{x}}_{j}}{\sqrt{\left(T-1\right)s_{x_{j}}^{2}}}\right)=z_{i}^{\prime }\cdot z_{j}^{\prime },$$

where $s_{x_{i}}^{2}$ is the unbiased sample variance of *x_i_* and $z_{i}^{\prime }$ is given by $z_{i}^{\prime }=\left(z_{i1}^{\prime },z_{i2}^{\prime },\ldots ,z_{iT}^{\prime }\right)$ with

$$z_{ik}^{\prime }=\frac{x_{ik}-{\bar{x}}_{i}}{\sqrt{\left(T-1\right)s_{x_{i}}^{2}}}=\frac{x_{ik}-{\bar{x}}_{i}}{\sqrt{\overset{T}{\underset{l=1}{\sum }}{\left(x_{il}-{\bar{x}}_{i}\right)}^{2}}}.$$

To compute **R** (formed by all pairwise correlation coefficients), we first compute $z_{i}^{\prime }$ for every node (pre-normalization; linear complexity) before computing $r_{i,j}=z_{i}^{\prime }\cdot z_{j}^{\prime }$ for every node pair (quadratic complexity), which saves one division per pair compared to computing *z_i_* and $\frac{z_{i}\cdot z_{j}}{T-1}$, respectively.

Depending on the processor's capabilities, AVX or SSE2 instructions are used for SIMD-based vectorization. SIMD instructions allow for data-level parallelism by carrying out the same operation on multiple data elements simultaneously, using 256-bit (AVX) or 128-bit (SSE2) wide SIMD registers. For implementation details see Loewe et al. (2014).

##### 2.1.2.4. Tetrachoric correlation estimation

Based on two binary variables *x*_*b*_ and *y*_*b*_, the tetrachoric correlation coefficient *r*_*t*_ (Pearson, 1900) estimates the correlation of the two latent continuous-valued variables *x*_*c*_ and *y*_*c*_, which are assumed to underlie *x*_*b*_ and *y*_*b*_. Originally, *r*_*t*_ has been devised for those cases in which *x*_*b*_ and *y*_*b*_ are observable while *x*_*c*_ and *y*_*c*_ are not. As recently proposed, it can also be used as a computationally efficient, although less accurate, alternative to Pearson's *r* as a functional connectivity estimate (Loewe et al., 2014). The use of *r*_*t*_ for this purpose requires data reduction in the temporal domain: each nodal time series is initially binarized based on its median (Loewe et al., 2013, 2014). Assuming bivariate normality, the correlation between two original (real-valued) time series can then be estimated very efficently based on the binarized time series by the tetrachoric correlation coefficient, exploiting SSE2 and the POPCNT instruction for SIMD-based vectorization for the necessary computations. If these instructions are not supported by the processor, an alternative, albeit less efficient, implementation based on a 16-bit lookup table can be used instead. For implementation details see Loewe et al. (2014). An additional advantage is that a binarized time series, being stored in the bits of integer variables, requires only a fraction of the memory that the original time series requires. This compression seems to render data locality optimization unnecessary for most data sets, presumably because the entire binarized time series data set can be stored in the CPU cache.

Note that the considerations made above for the half-stored scheme are also valid for the full-stored scheme. However, we decided to forego an actual implementation of this scheme within our framework at this point since it exhibits the highest memory requirements and a full-stored matrix representation is easily achieved based on already available software (for example, Matlab's corrcoef).

#### 2.1.3. Cache-based implementation

To accomodate situations where all matrix elements need to be traversed, but the order of traversal is essentially arbitrary, a *cache-based* storage scheme was additionally devised. In this scheme, upon construction, the matrix object is initialized such that a cache of user-specified size is maintained internally. While client code uses iterator functions to traverse the elements, the object internally fills its cache when necessary.

Since the order of traversal is determined by the object, both data locality (COBL) and multi-threading can be exploited when the cache is filled. Thus, similar to the half-stored scheme, data locality optimization and multi-threading can be implemented by the matrix object, which reduces the programmer's efforts when implementing applications, compared to the on-demand scheme. As mentioned above, for the on-demand scheme multi-threading has to be implemented by application code, and data locality optimization cannot be used at all, if inter-dependencies between matrix object implementation and client code are to be avoided. On the other hand, it is not possible to parallelize the traversal when using the cache-based variant, which could be a significant disadvantage in some situations.

### 2.2. Application: degree centrality

In this section, the task of node degree computation based on nodal time series data will serve as an example application to assess the performance of the different matrix object implementations with respect to both time and memory efficiency. The node degree, or degree centrality, is a simple graph-theoretical metric aimed at investigating the importance of individual nodes in a binary graph (Nieminen, 1974; Freeman, 1979). It is defined for each node as the number of other nodes to which it is connected. More formally, given a binary graph *G*_*B*_, the degree *k*_*i*_ of a node *i* is defined as $k_{i}=\overset{\left|N\right|}{\underset{j}{\sum }}b_{i,j}$, where *i, j* ∈ *N*, *i* ≠ *j*, and *N* is the set of nodes. In the neuroimaging literature, centrality measures have been used as a means to identify and analyze network hubs in the human brain (Buckner et al., 2009; Lohmann et al., 2010; Tomasi and Volkow, 2010, 2012; Wink et al., 2012; Zuo et al., 2012; Di Martino et al., 2013; van den Heuvel and Sporns, 2013; Binnewijzend et al., 2014; Markett et al., 2014, 2016; Schaefer et al., 2014).

#### 2.2.1. Programs

Combining the storage schemes *half-stored, on-demand*, and *cache-based* with the two functional connectivity estimates, Pearson's *r* and tetrachoric correlation coefficient *r*_*t*_, we arrive at six matrix object configurations. We will denote these by FCMAT/*s*/$\hat{\rho }$, where *s* indicates the storage scheme with *s* ∈ {half-stored, on-demand, cache-based}, and $\hat{\rho }$ indicates the functional connectivity estimate^2^ with $\hat{\rho }\in \left\{r,r_{t}\right\}$.

For typical fMRI data sets, data locality optimization turned out not to be beneficial if *r*_*t*_ is used as the functional connectivity estimate. Only for a very large number of points in time is a benefit observed. Presumably, this is because the binarized time series, due to efficient bitwise storage, take up far less memory than the original time series, thus enabling efficient CPU cache usage without additional optimizations. This is why the cache-based variant is not combined with *r*_*t*_ here. For the same reasons, data locality optimization is used for FCMAT/half-stored/*r*, but not for FCMAT/half-stored/*r*_*t*_.

The matrix object variants were implemented in C. The programs for degree computation based on the matrix object were also written in C, but Matlab integration is provided via MEX. A corresponding open source software package is available for download^3^. In addition, four programs for degree computation based on Matlab functions or Matlab-based third-party software have been created. These programs use corrcoef (Matlab built-in), corr (Matlab Statistics Toolbox), IPN_fastCorr, and IPN_calLCAM (from the Matlab toolbox “IPN_voxelGraph” by Xi-Nian Zuo available at Matlab File Exchange^4^) for matrix computation, respectively. Note that “IPN_voxelGraph” is now part of a new, much more extensive toolbox called “Connectome Computation System”^5^ (Xu et al., 2015).

In principle, each program first derives a weighted graph *G* (correlation matrix) from the data, then derives a binary graph *G*_*B*_ (adjacency matrix) from *G* based on a functional connectivity threshold, and finally determines the degree *k*_*i*_ of each node *i* in *G*_*B*_. Note that these basic steps are not necessarily performed explicitly.

The programs based on corrcoef, corr, and IPN_fastCorr use a full-stored matrix representation as they first compute an *N* × *N* correlation matrix. This matrix is thresholded to derive a binary adjacency matrix. Then, degrees are obtained by summation over rows (or columns) of the adjacency matrix.

The programs using FCMAT/half-stored/^*^ first create the matrix object, which, upon creation, computes and internally stores the upper triangle of the correlation matrix based on the input data. In doing so, multi-threading, data locality optimization, and vectorization are used. Data locality optimization is used only in combination with Pearson's *r* for the reasons stated above. The matrix elements are then traversed using the object's accessor method (traversal is parallelized using multiple threads). Upon access, the appropriate pre-computed element is returned by the matrix object. If an element exceeds the threshold, the degree of the corresponding nodes is incremented.

The programs using FCMAT/on-demand/^*^ first create the matrix object, which, upon creation, pre-normalizes or binarizes the input data. The matrix elements are then traversed using the object's accessor method (traversal is parallelized using multiple threads). Upon access, the appropriate element is computed, and returned by the matrix object. If an element exceeds the threshold, the degree of the corresponding nodes is incremented. Neither the correlation matrix nor the adjacency matrix is explicitly stored.

The program using FCMAT/cache-based/*r* first creates the matrix object, which, upon creation, initializes the internal cache. The matrix elements are then traversed using the iterator functions. When the next matrix element is requested during traversal, the matrix object checks if the next element is in the cache and retrieves it from there, if that is the case. If not, the cache is filled by computing the next “tile of matrix elements” exploiting multi-threading, data locality optimization, and vectorization in the process. The matrix element along with its coordinates is then returned by the matrix object. Thresholding and incrementation of the appropriate degrees are conducted during traversal in the same way as for the other variants, with the notable exception that the traversal (and hence also thresholding and degree incrementation) is not parallelized.

The program using IPN_calLCAM does not follow any of the schemes described above. Based on a pre-defined correlation threshold, it employs a block-wise approach to construct a sparse adjacency matrix directly from the data (Zuo et al., 2012). The adjacency matrix is efficiently stored using Matlab's sparse matrix functionality. Explicit storage of the full correlation matrix is thus avoided, while memory requirements during adjacency matrix construction depend on the selected block size, and memory requirements for the final adjacency matrix depend on its sparsity. To efficiently compute the correlation values for each block, IPN_calLCAM internally uses IPN_fastCorr. Note that IPN_calLCAM is not applicable to analyses that use the (weighted) connectivity matrix rather than the adjacency matrix.

#### 2.2.2. Benchmarks

Benchmarks were conducted using Matlab (R2011b) on two machines, a desktop computer with an Intel Core i7-3960X CPU (3.30 GHz, 6 cores, hyper-threading disabled) and 64 GB of main memory running Linux (openSUSE 13.1, Kernel 3.11), and a server with two Intel Xeon E5-2697 v2 CPUs (2.7 GHz, 12 cores, hyper-threading enabled) and 256 GB of main memory running Linux (Ubuntu 12.04.5 LTS, Kernel 3.13). The C/MEX routines that are part of the programs that use the matrix object were compiled using the GNU C compiler gcc (optimization level 3; version 4.8.1 and 4.6.3 on the desktop and the server system, respectively). To assess the memory usage of the programs, we used the function monMem^6^, which, in turn, uses the Linux proc file system to monitor Matlab's resident memory size during program execution.

Three input data sets with a different number of nodes (60,000, 120,000, and 240,000) and *T* = 256 points in time were generated using pseudo-random single-precision floating point numbers. Storage of the full matrix (assuming 4 byte per element) would require 13.4, 53.6, and 214.6 GiB of memory for 60,000, 120,000, and 240,000 nodes, respectively. The correlation threshold was chosen such that the density of the resulting binary graph was approximately 0.01. For IPN_calLCAM, we used 10 (for the data sets with 60,000 and 120,000 nodes) and 25 blocks (for the data set with 240,000 nodes). The number of threads was varied between 1 and 6 on the desktop computer and between 1 and 48 on the server. For the programs based on the FCMAT variants the number of threads was controlled via the corresponding parameter. For the programs based on corrcoef, corr, IPN_fastCorr, and IPN_calLCAM the number of threads was controlled using the Matlab function maxNumCompThreads.

## 3. Results

To assess the performance of the different FCMAT variants, we used the computation of node degrees based on nodal time series data as an example application. Experiments were conducted on the two machines described above. Note that, depending on the number of nodes of the input data set and the available main memory, some tests could not be conducted due to insufficient memory on the respective systems. In correspondence with the number of logical processors on each system, the number of threads was varied between 1 and 6 on the desktop computer and between 1 and 48 on the server.

**Figure 1:**
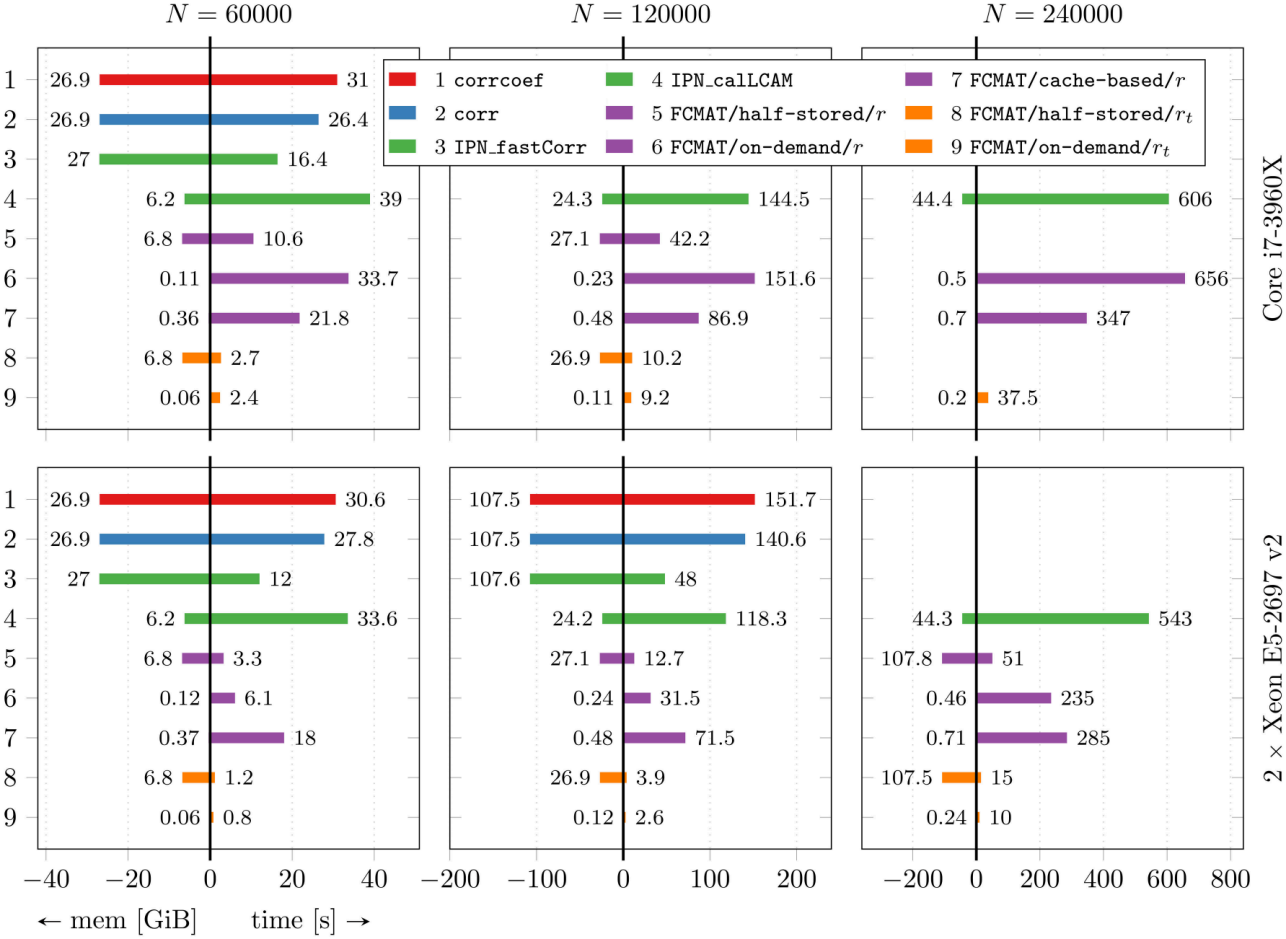
**Performance comparison with respect to time and memory efficiency**. The compared programs for degree computation are based on corrcoef (1), corr (2), IPN_fastCorr (3), IPN_calLCAM (4) and the proposed functional connectivity matrix object FCMAT using the half-stored (5 and 8), the on-demand (6 and 9) and the cache-based variant (7). The programs 1-7 use Pearson's *r* as a functional connectivity estimate; the programs 8 and 9 use the tetrachoric correlation coefficient *r*_*t*_. Comparisons were conducted on two machines, a desktop computer with an Intel Core i7-3960X CPU and 64GB of main memory, and a server with two Intel Xeon E5-2697 v2 CPUs and 256GB of main memory. The number of threads was varied between 1 and 6 on the desktop computer and between 1 and 48 on the server. For each program, only the best result is reported, i.e., the result based on the number of threads for which the elapsed time was shortest for that program. See Figure 2 for more detailed results regarding the performance gained by each program through multi-threading. The reported results are averages from 10 runs. For details see text. The number of timepoints *T* was fixed at *T* = 256. *N*: number of nodes; mem [GiB]: peak memory in GiB; time [s]: elapsed time in seconds.

**Figure 2:**
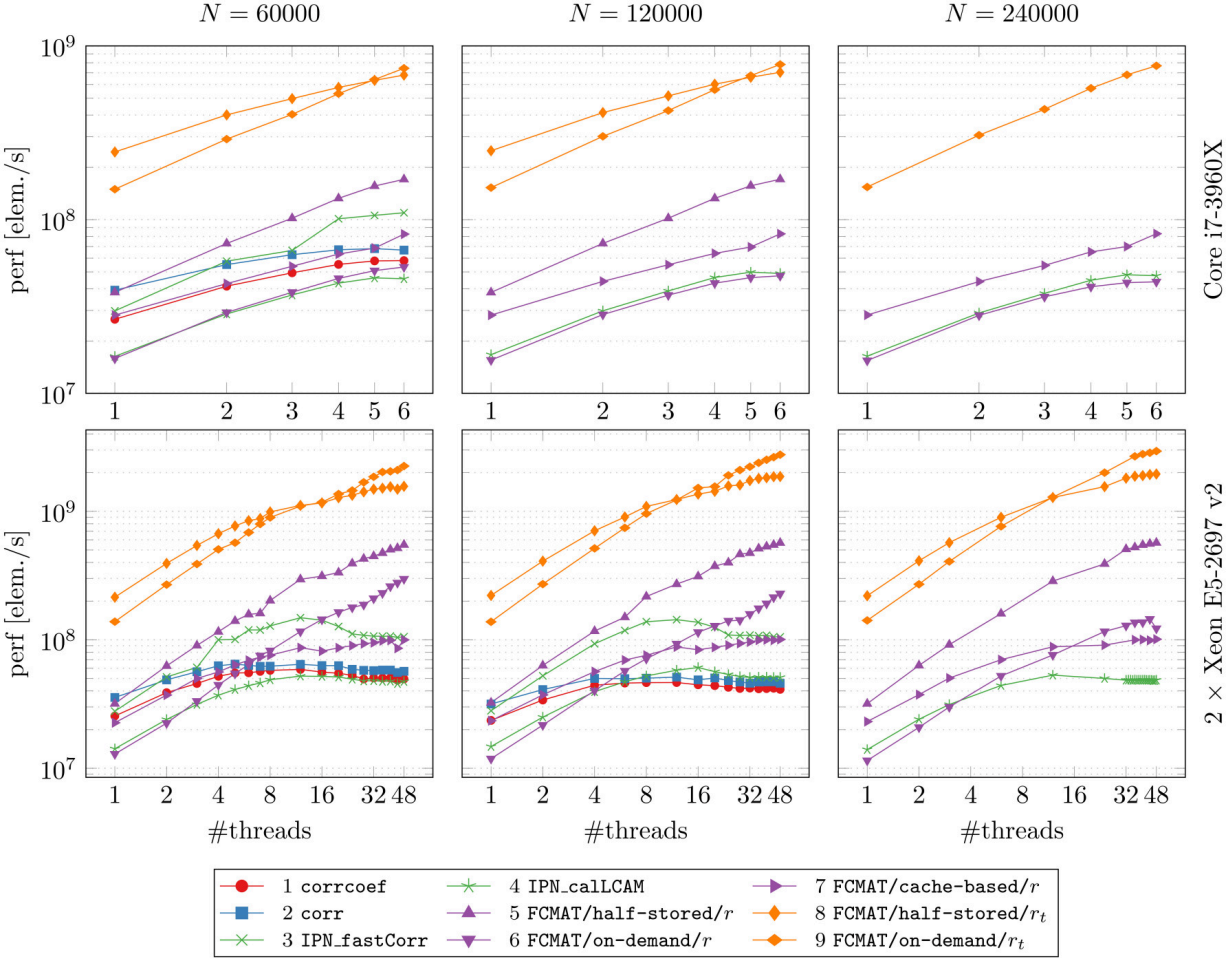
**Performance gained through multi-threading**. The compared programs for degree computation and the two machines on which the comparisons were conducted are the same as in Figure 1. The reported results are averages from 10 runs on each machine. The number of timepoints *T* was fixed at *T* = 256. *N*: number of nodes; perf [elem./s]: performance in number of computed elements per second.

**Figure 3:**
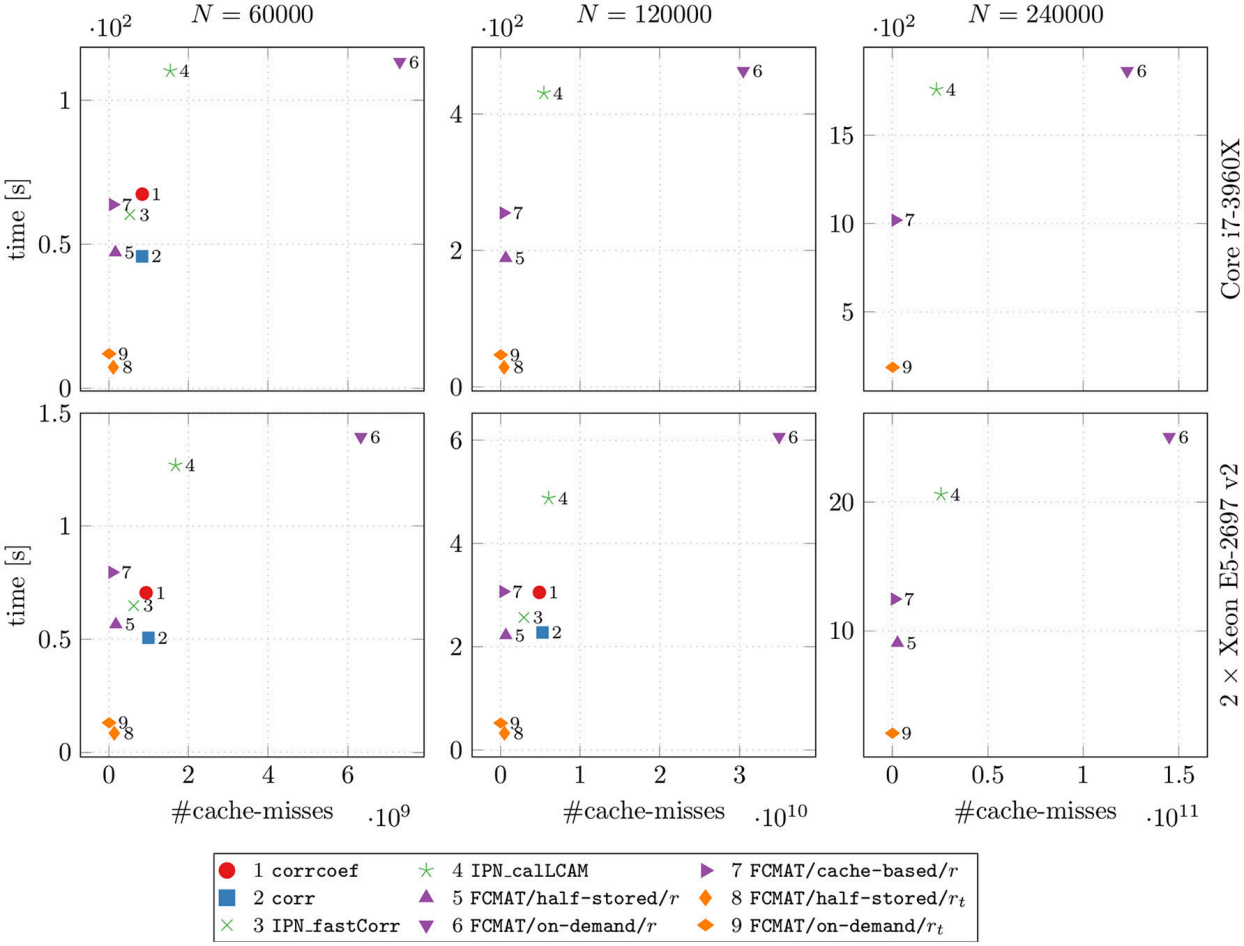
**Speed vs. cache misses**. The compared programs for degree computation and the two machines on which the comparisons were conducted are the same as in Figures 1 and 2. The reported results are averages from 10 runs on each machine. The number of timepoints *T* was fixed at *T* = 256. *N*: number of nodes; time [s]: elapsed time in seconds.

In Figure 1, the main results regarding memory requirements and computation time are illustrated. Here, only the best result is reported for each program, i.e., the result based on the number of threads for which the elapsed time was shortest for that program. This seemed the most appropriate since some programs continually gained performance from additional threads, while for other programs additional threads turned out to be detrimental to their performance after a certain optimal number of threads was exceeded. The performance gained by each program through multi-threading and the cache effectiveness in terms of cache misses are illustrated in Figures 2 and 3, respectively.

### 3.1. Memory requirements and maximum performance

The benchmarks showed that the programs based on corrcoef (1), corr (2), and IPN_fastCorr (3) had the highest memory requirements of all programs (Figure 1), which was expected because they employ full matrix storage. However, they used more memory than expected (Table 1). More specifically, the peak memory of these three programs was about two times higher than expected. Instead of approximately 4(*N*^2^ + *NT*) bytes it was about twice that much, for example, approximately 27 GiB instead of the expected 13.5 GiB for *N* = 60,000. Of these three programs, IPN_fastCorr executed fastest, sometimes up to 3× faster than corrcoef, while corr was only slightly faster than corrcoef (Figure 1).

**Table 1:** **Expected and measured memory usage**.

**Name**	**Memory Usage (expected)**	**Memory Usage (measured)**					
	**# bytes**	***N* = 6 · 10^4^**	**12 · 10^4^**	**24 · 10^4^**	**6 · 10^4^**	**12 · 10^4^**	**24 · 10^4^**
corrcoef	4(*N*^2^ + *NT*)	13.47	53.76	214.81	26.94	107.52	
corr	4(*N*^2^ + *NT*)	13.47	53.76	214.81	26.94	107.52	
IPN_fastCorr	4(*N*^2^ + *NT*)	13.47	53.76	214.81	26.99	107.63	
IPN_calLCAM	? + 4*NT*				6.23	24.21	44.33
FCMAT/half-stored/*r*	2*N*(*N* − 1) + 8*NT*	6.82	27.05	107.75	6.83	27.06	107.75
*r*_*t*_	$2N\left(N-1\right)+4NT+\frac{B}{8}N\left\lceil T/B\right\rceil$	6.76	26.94	107.52	6.77	26.94	107.52
on-demand/*r*	8*NT*	0.11	0.23	0.46	0.12	0.24	0.46
*r*_*t*_	$4NT+\frac{B}{8}N\left\lceil T/B\right\rceil$	0.06	0.12	0.24	0.06	0.12	0.24
cache-based/*r*	4*C*^2^ + 8*NT*	0.36	0.48	0.71	0.37	0.48	0.71

The memory requirements of IPN_calLCAM (4) depend on the number of blocks *S*, so that it is possible to reach lower memory requirements at the cost of increased computation time (due to additional overhead) by choosing a greater number of blocks. Using *S* = 10, *S* = 10, and *S* = 25, the program peaked at approximately 6.2, 24.3, and 44.4 GiB for *N* = 60,000, *N* = 120,000, and *N* = 240,000, respectively. For *N* = 60,000, IPN_calLCAM executed about 10–30% slower than corrcoef (depending on the system), while it was about 20% faster for *N* = 120,000 on the server (Figure 1).

The programs based on FCMAT/half-stored/*r* (5) and FCMAT/half-stored/*r*_*t*_ (8) showed the expected memory requirements, which were significantly reduced compared to the programs based on full storage, but still very high. They peaked at about about 7, 27, and 108 GiB for *N* = 60,000, *N* = 120,000, and *N* = 240,000, respectively. Here, the expected memory requirements were computed as

$$\underset{\text{resulting matrix}}{\underset{︸}{2N(N-1)}}+\underset{\text{original data}}{\underset{︸}{4NT}}+\underset{\text{normalized data}}{\underset{︸}{4NT}}\;\text{bytes}$$

for FCMAT/half-stored/*r*, and

$$\underset{\text{resulting matrix}}{\underset{︸}{2N(N-1)}}+\underset{\text{original data}}{\underset{︸}{4NT}}+\underset{\text{binarized data}}{\underset{︸}{\frac{B}{8}N\left\lceil T/B\right\rceil }}\;\text{bytes}$$

where *B* is the number of bits per integer variable, for FCMAT/half-stored/*r*_*t*_ (Table 1). Depending on the system and the number of nodes, FCMAT/half-stored/*r* and FCMAT/half-stored/*r*_*t*_ ran between 3 and 12× and 11 and 39× faster than corrcoef (Figure 1).

The memory requirements of the programs based on FCMAT/on-demand/*r* (6) and FCMAT/on-demand/*r*_*t*_ (9) remained below 0.5 GiB for all three data sets. Specifically, the former peaked at approximately 0.12, 0.23, and 0.46 GiB corresponding to the expected 4*NT* (original data) + 4*NT* (normalized data) bytes, while the latter peaked at approximately 0.06, 0.12, and 0.24 GiB, corresponding to 4*NT* (original data) + $\frac{B}{8}N\left\lceil T/B\right\rceil$ (binarized data) bytes, for *N* = 60,000, *N* = 120,000, and *N* = 240,000, respectively. Depending on the system and the number of nodes, FCMAT/on-demand/*r* executed 1.8–3.6× slower than the corresponding *half-stored* variant FCMAT/half-stored/*r*, while FCMAT/on-demand/*r*_*t*_ executed up to 1.5× faster than its *half-stored* pendant FCMAT/half-stored/*r*_*t*_ (Figure 1).

The program based on FCMAT/cache-based/*r* (7) peaked at approximately 0.36 (*N* = 60,000), 0.48 (*N* = 120,000), and 0.71 GiB (*N* = 240,000) corresponding to the expected 4*C*^2^ + 8*NT* bytes (using a cache tile size of *C* = 8192). On the desktop, depending on the number of nodes, FCMAT/cache-based/*r* executed about 1.6–1.9× faster than the corresponding on-demand variant and about 2.1× slower than the corresponding half-stored variant. On the server, depending on the number of nodes, it executed about 1.2–3× slower than the on-demand variant and about 5.5× slower than the half-stored variant (Figure 1).

### 3.2. Performance gain through multi-threading

After reaching a performance maximum at a certain number of threads, the programs using corrcoef, corr, IPN_fastCorr, and IPN_calLCAM exhibited stagnating and even decreasing performance upon adding more computational threads (Figure 2, especially on the server). A similar observation can be made for FCMAT/cache-based/*r*, although performance seems to reach a plateau state of maximum performance, such that additional threads do not improve performance further but no performance decrease is observed either. On a related note, FCMAT/cache-based/*r* is significantly faster than FCMAT/on-demand/*r* for smaller numbers of threads, but this advantage is lost in the course of more threads being added (Figure 2, especially on the Xeon system). This is because FCMAT/on-demand/*r* sustains a relatively constant high performance gain per thread over the entire range of the number of threads. A similar observation can be made for FCMAT/on-demand/*r*_*t*_ and FCMAT/half-stored/*r*_*t*_ (Figure 2, both systems).

### 3.3. Cache misses

To assess how well the attempted data locality optimization worked (especially with regard to FCMAT/cache-based/r), we compared the programs' computation time with the number of cache misses (Figure 3). Note that these measurements were conducted using only one thread.

Comparing the programs using FCMAT/*/r, the on-demand variant had the greatest number of cache misses and correspondingly exhibited the lowest performance (with respect to computation time). The other two variants exhibited a similar number of cache misses (both had significantly fewer cache misses than the on-demand variant), but the half-stored variant still ran faster than the cache-based variant, while both ran significantly faster than the on-demand variant. Similarly, the programs using corrcoef and corr exhibited a similar number of cache misses, but the latter outperformed the former. Furthermore, corrcoef was about as fast as FCMAT/cache-based/r and corr was about as fast as FCMAT/half-stored/r, although corrcoef and corr exhibited more cache misses than the other two. The program using IPN_fastCorr exhibited a lower number of cache misses than both corrcoef and corr but higher than FCMAT/half-stored/r and FCMAT/cache-based/r. Its performance ranked between that of corrcoef and corr. The number of cache misses for IPN_calLCAM was comparable with that of corrcoef and corr, but it was significantly slower than both.

## 4. Discussion

Dense connectomes allow for spatially more fine-grained connectivity analyses, but are associated with significant computational demands (van den Heuvel et al., 2008; Hayasaka and Laurienti, 2010; de Reus and Van den Heuvel, 2013; Fornito et al., 2013). Available computing resources are often insufficient to meet these demands, so that dense connectome analyses become infeasible (e.g., Smith et al., 2014). In an attempt to address this issue, we presented an object-based functional connectivity matrix representation (FCMAT) and corresponding implementation variants, tailored for the analysis of dense functional connectomes. Based on theoretical considerations and benchmarks, different implementation variants of this object and four additional programs (based on available Matlab functions and Matlab-based third-party software) were compared with regard to their computational efficiency in terms of both memory requirements and computation time.

The most memory-efficient FCMAT variant avoids explicit matrix storage by computing matrix elements *on demand* based on the underlying time series data (FCMAT/on-demand/^*^). Since, in the considered application domain, the number of nodes is much greater than the number of scans, the memory requirements of a dense connectome are effectively reduced by orders of magnitude compared to explicit storage of the connectivity matrix.

However, explicit storage has an advantage over on-demand computation when it comes to computation time because data locality optimization can be employed. Accordingly, the implementation using explicit matrix storage, FCMAT/half-stored/^*^, is the fastest of the presented variants. It is also faster than the alternatives based on explicit storage (full-stored), corrcoef (Matlab built-in), corr (Matlab Statistics Toolbox), and IPN_fastCorr (from the Matlab toolbox “IPN_voxelGraph” by Xi-Nian Zuo)^7^. Of these, IPN_fastCorr was significantly faster than the other two. Regarding memory requirements, FCMAT/half-stored/^*^ has an advantage over the full-stored programs because it exploits matrix symmetry in order to save memory. Nevertheless, the memory requirements of FCMAT/half-stored/^*^ are still too high for many applications.

In addition to FCMAT/half-stored/^*^ and FCMAT/on-demand/^*^, a third, cache-based variant, FCMAT/cache-based/*r*, was implemented in an attempt to combine the advantages of the first two. Regarding time efficiency, this variant is at an advantage over the on-demand variant, in that it can use data locality optimization, which is reflected in the observed reduction in cache misses. It is, however, at a disadvantage compared to the half-stored variant, because of the overhead that results from maintaining the cache. Regarding memory efficiency, the cache-based scheme requires far less memory than the half-stored scheme although not quite as little as the on-demand scheme (assuming a sensible choice for the cache size). Depending on the application, the cache-based scheme may thus provide a reasonable compromise between explicit storage and on-demand computation, by being more memory-efficient than the former, but faster than the latter. However, while the computation of the matrix elements by the matrix object can be parallelized (during cache fills), the traversal of these elements by the client code can not be parallelized. If additional, potentially expensive, computations need to be conducted during matrix traversal, this drawback can tip the balance in favor of one of the other implementations for some applications (see Amdahl, 1967). In this context, the number of cores of the system also needs to be taken into account. As the benchmarks illustrate, the on-demand variant should be chosen over the cache-based variant on a system with many cores because the impact of this issue increases with the degree of parallelization.

Considering the example application used for benchmarking, each of the created programs for node degree computation (implicitly or explicitly) derives a binary adjacency matrix from the connectivity matrix based on a threshold. Depending on the thresholding scheme used (e.g., based on graph density), an additional run through the data may be necessary to determine the threshold in terms of absolute correlation before the degree map can be computed. For those programs that do not operate on the explicitly stored connectome (IPN_calLCAM, FCMAT/on-demand/^*^ and FCMAT/cache-based/r), this entails that the correlation values need to be computed twice. This example makes clear that, especially in situations where the matrix or connectome will be used more than once (e.g., when subjected to multiple analyses), explicit storage should be used if it is affordable (with respect to the available main memory) in order to save computation time.

Although the present programs provide a highly efficient way of conducting analyses, there are some limitations that should be noted. First, in cases of extremely large multi-subject data sets (in terms of number of subjects or scans), too much memory may still be required for these methods to be applied because the time series data of all subjects could already be too large to be stored in main memory. In some cases, the analysis procedure could be adapted to hold only a sufficiently small partition of the data in memory at any given time. Second, the current implementation only supports Pearson's *r* and *r*_*t*_. In the case of dense connectomes, the number of nodes and therefore edges is a lot higher than the number of scans, so that the underlying population correlation ρ cannot be estimated very accurately using *r* or *r*_*t*_ (e.g., Varoquaux and Craddock, 2013). The extension of FCMAT to support other, possibly better estimates is a subject for future work. Finally, while FCMAT provides the basis for the efficient analysis of dense connectomes, the tools for the analyses themselves (for example, graph-theoretical analyses or statistical inference), still need to be implemented (on top of it), which, depending on the analysis in question, may prove to be a lot more difficult.

To summarize, when considering the computational burden of dense functional connectome analysis, the manner in which connectivity matrices are represented plays an important role. We show here that a set of complementary implementation variants of an object-based matrix representation (FCMAT) provide a highly efficient foundation for dense connectome analysis. If affordable in terms of available memory, explicit matrix storage should be used, since it provides the best performance in terms of CPU time. However, if its memory requirements render the use of explicit storage infeasible, on-demand computation or cache-based iteration provide memory-efficient alternatives. In particular, the on-demand and cache-based implementation variants allow for the analysis of larger data sets on commonly available hardware, which may not have been possible before, based on explicit storage. With the ever-growing need for maximal spatial precision and resolution among large sets of subjects in fMRI connectivity analyses, the development of efficient tools such as these is paramount to the advancement of our understanding of the human brain.

## Author contributions

KL conceived of the study. KL and CB wrote the software. KL carried out the benchmarks. KL wrote the manuscript. SD, MS, RK, and CB edited the manuscript. All authors read and approved the final manuscript.

## Funding

This work was in part supported by Deutsche Forschungsgemeinschaft SFB 779 (A14N).

### Conflict of interest statement

The authors declare that the research was conducted in the absence of any commercial or financial relationships that could be construed as a potential conflict of interest.

## Footnotes

^1^A single instruction can be applied to multiple data in parallel using CPU instruction set extensions such as SSE2 (Streaming SIMD Extensions, version 2) or AVX (Avanced Vector eXtensions).

^2^The symbol ρ was chosen because the functional connectivity estimates that are used here, *r* and *r*_*t*_, are, in fact, correlation estimates (estimating a hypothetical population correlation ρ). This involves the simplistic assumption of time series stationarity (Hutchison et al., 2013; Zalesky et al., 2014) of the involved time series and also the assumption of bivariate normality between every two time series (Hlinka et al., 2011).

^3^http://www.kristianloewe.com/software and https://github.com/kloewe/conan.

^4^http://www.mathworks.com/matlabcentral/fileexchange/32553-ipn-voxelgraph.

^5^https://github.com/zuoxinian/CCS.

^6^https://github.com/kloewe/util-m.

^7^http://www.mathworks.com/matlabcentral/fileexchange/32553-ipn-voxelgraph.
